# Intradiscal Pressure Changes during Manual Cervical Distraction: A Cadaveric Study

**DOI:** 10.1155/2013/954134

**Published:** 2013-08-20

**Authors:** M. R. Gudavalli, T. Potluri, G. Carandang, R. M. Havey, L. I. Voronov, J. M. Cox, R. M. Rowell, R. A. Kruse, G. C. Joachim, A. G. Patwardhan, C. N. R. Henderson, C. Goertz

**Affiliations:** ^1^Palmer Center for Chiropractic Research, 741 Brady Street, Davenport, IA 52803, USA; ^2^Hines VA Hospital, 5000 South 5th Avenue, Hines, IL 60141, USA; ^3^Cox Chiropractic Medicine, Inc., 3125 Hobson Road, Fort Wayne, IN 46805, USA; ^4^Chiropractic Care, Ltd., 2417 183rd Street, Homewood, IL 60430, USA; ^5^Aaron Chiropractic Clinic, 3476 Stellhorn Road, Fort Wayne, IN 46815, USA; ^6^Loyola University Stritch School of Medicine, 2160 S. First Avenue, Maywood, IL 60153, USA; ^7^Henderson Technical Consulting, 5961 Broken Bow Lane, Port Orange, FL 32127, USA

## Abstract

The objective of this study was to measure intradiscal pressure (IDP) changes in the lower cervical spine during a manual cervical distraction (MCD) procedure. Incisions were made anteriorly, and pressure transducers were inserted into each nucleus at lower cervical discs. Four skilled doctors of chiropractic (DCs) performed MCD procedure on nine specimens in prone position with contacts at C5 or at C6 vertebrae with the headpiece in different positions. IDP changes, traction forces, and manually applied posterior-to-anterior forces were analyzed using descriptive statistics. IDP decreases were observed during MCD procedure at all lower cervical levels C4-C5, C5-C6, and C6-C7. The mean IDP decreases were as high as 168.7 KPa. Mean traction forces were as high as 119.2 N. Posterior-to-anterior forces applied during manual traction were as high as 82.6 N. Intraclinician reliability for IDP decrease was high for all four DCs. While two DCs had high intraclinician reliability for applied traction force, the other two DCs demonstrated only moderate reliability. IDP decreases were greatest during moving flexion and traction. They were progressevely less pronouced with neutral traction, fixed flexion and traction, and generalized traction.

## 1. Introduction

Neck pain and neck-related shoulder and arm pain are a major health problem in Western societies [1–5]. Symptoms may include pain, tingling, numbness, stiffness, loss of coordination or physical strength, skin discoloration, and temperature differences located in the neck, shoulder, arm, elbow, wrist, hand, and/or fingers. These complaints cause discomfort and may lead to severe long-term pain and physical disability creating an economic burden due to work absences and healthcare costs [1]. In 2003 the 12-month prevalence of neck and shoulder pain in The Netherlands was estimated at 31.4% and 30.3%, respectively [6]. In 2008, approximately 6% of US adults reported an ambulatory visit for a primary diagnosis of a back or neck condition (13.6 million). Between 1999 and 2008, the mean inflation-adjusted annual expenditures on medical care for these patients increased by 95% (from $487 to $950); most of the increase was accounted for by increased costs for medical specialists, as opposed to primary care physicians. During the study period, the mean inflation-adjusted annual expenditures on chiropractic care were relatively stable. Physical therapy was the most costly service overall [7]. 

Spinal manipulation is used by doctors of chiropractic (DC), osteopathic physicians, and physical therapists to treat musculoskeletal disorders [8–12]. While spinal manipulation has been shown to be effective in some studies [13], and researchers have performed experimental studies with humans and animals [14–21], the exact mechanisms behind these techniques are not fully understood [22]. 

A form of chiropractic manipulation performed using a specially designed table that incorporates traction called manual cervical distraction (MCD), or flexion distraction, was developed by Cox  [23]. Several case studies have reported clinical improvement of patients with neck pain [24–28]. MCD is hypothesized to create intersegmental motion at a targeted segment under the application of traction via a load localizing hand contact utilizing a treatment table [23]. The effects of traction for the cervical spine may include separation of vertebrae, reduction of intradiscal pressure (IDP), facet joint separation, increase of intervertebral foramen, and soft tissue stretching [29–31]. 

The resulting traction-induced intersegmental motion is thought to open the intervertebral foramen and decrease intradiscal pressures (IDPs). 

Li et al. [32] and Wu et al. [33] used a materials testing system to simulate cervical high velocity low amplitude spinal manipulation (HVLA SM) on human cervical cadaveric specimens. They reported IDP decreases during the traction phase prior to delivering HVLA and IDP increases during the rotational thrust manipulation. However, the clinical application of these findings is unclear because both Li and Wu performed their simulations with material testing systems; substantially different procedures than those used by clinicians. By contrast, the MCD procedure used in the present study is widely used. Sixty-four percent of doctors of chiropractic (DCs) treat neck pain with this method [34]. 

The objectives of this study were; in unembalmed cadavers with intact head, neck, and trunk: (1) measure IDP in the lower cervical spine (C4-C5, C5-C6, C6-C7, and C7-T1) and (2) during the MCD procedure performed by study DCs, measure the magnituded and reliability of applied forces.

## 2. Materials and Methods

**Figure 1:**
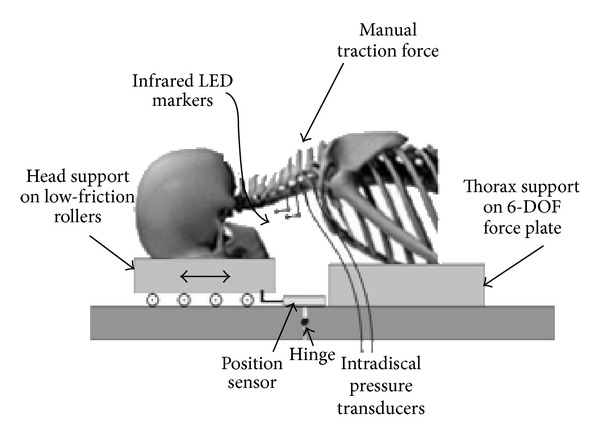
A schematic diagram of the experimental setup.

A specially modified treatment table incorporated a multicomponent (3 forces and 3 moments) force plate (Model number 2850-06, Bertec, Inc., Columbus, OH) into the thorax section of the table to which the specimen torso was mounted (Figure 1). The head support of the table allowed linear motion to create traction of the specimen's cervical spine, flexion motion of the head, and locking of the head support at a given flexion angle. 

### 2.1. Specimens

**Figure 2:**
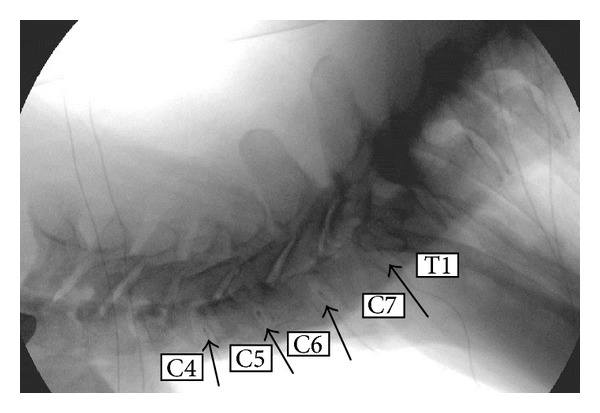
A videofluoroscopic image of the cervical spine with IDP sensors in the nucleus of C4-5, C5-6, C6-7, and C7-T1.

Nine fresh-frozen cadavers with intact head, neck, and trunk with shoulders were procured from approved tissue banks and stored in freezers at −20°C. Radiographs were taken to exclude severe degeneration, trauma, tumor, or significant osteoporosis.  Figure 2 is a static video fluoroscopic image showing (see arrows) the location of pressure transducers in the nucleus of the C4-C5, C5-C6, C6-C7, and C7-T1 intervertebral discs. Intervertebral discs were graded from the static video fluoroscopic images by three independent observers using disc height measurements [35, 36]. Demographics of the specimens are provided in Table 1.

Pressure sensors (Model 060, Precision Measurement, Inc., Ann Arbor, MI) were calibrated using a hand-held pressure calibration device (Model number HTP1, Druck, Ltd., Leicester, UK). Pressure calibrations were linear, and a first-order polynomial function was used to describe each calibration. Specimens were thawed at room temperature. Pressure transducers were inserted through anterior approach into the nucleus pulposus of C4-C5, C5-C6, C6-C7, and C7-T1. The transducers were inserted through a 14-gauge cannula into each disc nucleus under video fluoroscopy guidance (OEC-9800, GE Healthcare Systems, Waukesha, WI). 

After the sensors were inserted, the cadaver was placed in a prone position with the head resting on the moveable head support and the thorax resting on the fixed section of the table, which was mounted on the force plate. The thorax was rigidly secured to the table section and underlying force plate by Velcro straps. The head and upper cervical spine were positioned on the moveable headpiece. The thorax was positioned on the middle section of the table with the cervical spine between the cervical headpiece and thoracic section of the table. This allowed manual contact of the cervical and upper thoracic spine vertebrae for distraction in neutral position or in a flexion posture of the head.

 MCD was performed on the cadaver spines by three field clinicians and one academic/research clinician. All four clinicians are considered experts in delivering MCD. 

**Figure 3:**
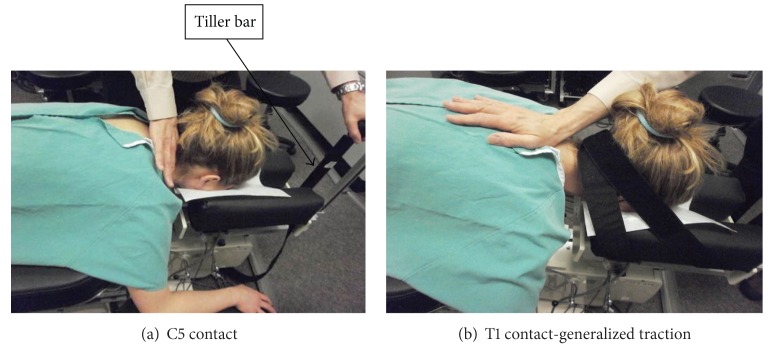
Photographs showing hand contact on a patient during MCD procedures.

**Figure 4:**
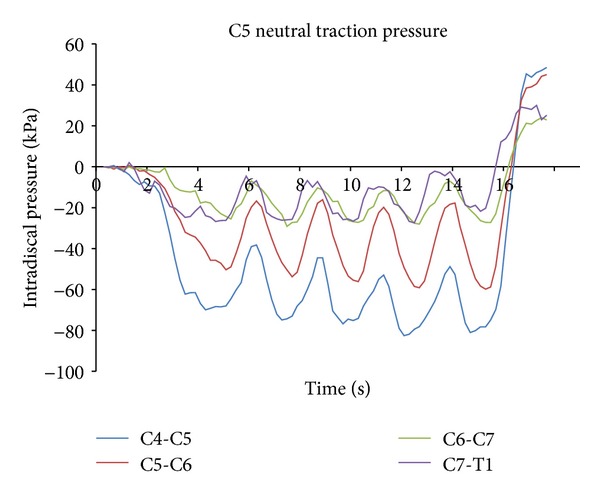
A typical graph showing changes in IDP as a function of the duration of MCD.

The MCD treatment protocol used was as follows. The web of the hand between thumb and index finger was placed on the spinous process and lamina above the segment to be distracted (Figure 3(a)). A controlled cephalad distraction was therefore applied to the vertebral segment by combined hand contact and headpiece motion of the table in the longitudinal direction of the spine. The distraction along the length of the spine was applied in three twenty-second-distraction sessions. During each twenty-second session there were five loading-unloading distraction cycles (Figure 4). These distraction sessions were applied at the C5 and C6 hand contact locations. 

The cervical headpiece of the table was then placed in a fixed flexion angle of 15 degrees, and the MCD procedure was repeated while the cervical head piece of the table moved in flexion and slid longitudinally. The cervical headpiece of the table was allowed to move in flexion freely while at the same time sliding on the cranial caudal axis to create traction. Thus the clinician was moving the head piece in flexion and traction simultaneously, and the cervical spine was subjected to flexion and traction movements simultaneously.

In another procedure, occipital restraints were placed on the cadaver skull and the doctor's hand contacted the vertebral arch at the T1 level with the thenar eminence of the hand contacting the spinous process at T1 level (Figure 3(b)). A generalized traction was then applied to the entire cervical spine by moving the table's tiller bar in the cephalad (superior) direction. This procedure was repeated with 15 degrees of flexion of the headpiece.

Custom data collection software was developed using a TestPoint programming environment (TestPoint v7, Measurement Computing, Inc., Norton, MA). The software allowed data collection from the pressure transducers and force plate simultaneously. Data were collected through an analog to digital convertor (Model NI-6225, National Instruments, Inc., Austin TX) at a sampling rate of 150 Hz. When data collection was complete, it was displayed in graphical form and imported into MS Excel.

### 2.2. Data Processing/Reduction

 Custom written macros in MS Excel identified the beginning and the peak force application for the 3 sets of 5 force application cycles. The IDPs were recorded at the beginning of the force application and at the peak of force application for all five cycles in each set. The mean of the 15 cycles was calculated. 

### 2.3. Statistical Analysis

Descriptive statistics (Systat v10.2, Systat Software, Inc., Chicago, IL) in terms of mean and standard deviation were computed for the changes in IDP and the forces of all measured data. Intraclass correlation coefficients (ICCs) were calculated for each clinician to evaluate intraclinician reliability across the three repititions of applied force.

## 3. Results

Study Chiropractor 1 (DC1) performed MCD on eight out of nine specimens, DCs 2 and 4 performed MCD on four out of nine specimens, and DC3 performed MCD on three out of nine specimens. IDP data could not be obtained on one of the nine specimens (DCs 1, 3, and 4 performed on this specimen) due to equipment technical difficulties. Thus IDP data and the force data had a different number of observations. Intervertebral discs were graded from the static video fluoroscopic images by three independent observers. Based on the disc height classification, most of the discs (34 out of 36) were of Grade I, one of Grade II, and one of Grade III degeneration (Table 2).

**Figure 5:**
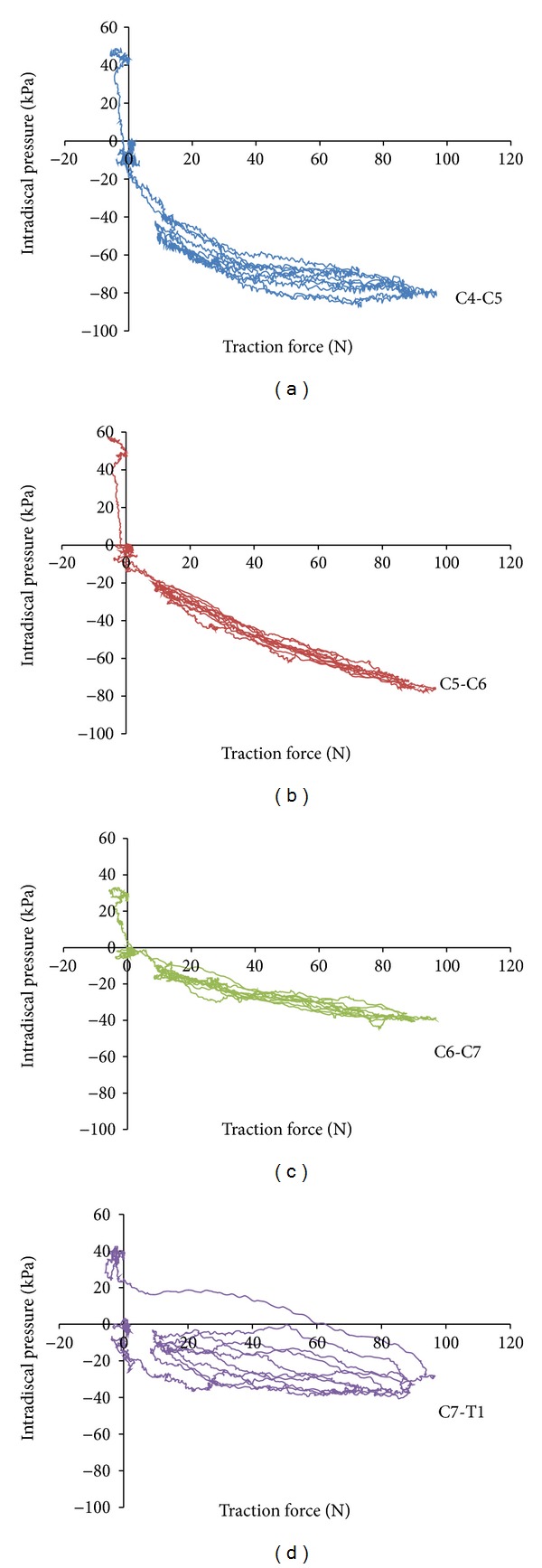
Pressure-force graphs—C6 manual contact with neutral head position.

Figure 4 shows typical IDP graphs at each of the lower cervical discs (C4-C5, C5-C6, C6-C7, and C7-T1) as a function of the duration of treatment, demonstrating the decrease in IDP as the DC applied MCD during the five loading/unloading cycles in a given session.  Figure 5 presents a typical graph showing the changes in IDP as a function of traction force (as measured by the force plate under the thorax support). 

Table 2 gives the mean and SD of IDP changes under different traction conditions for all four DCs. Varying magnitudes of IDP decreases were observed across the different DCs, contact location, and traction procedure in different positions of the head piece.  Table 3 gives the mean and SD of the applied forces for the four DCs under different traction conditions.  Table 4 provides intraclass correlation coefficients for assessing IDP and traction force intraclinician reliability during the three repetitions of MCD.

## 4. Discussion

Decreased IDP is thought to allow retraction of the prolapsed disc, contributing to improved solute and nutrient transport, and altering the chemical environment of nociceptors in the outer annular layers of the disc [29]. Manually localized lumbar distraction has already been shown to decrease IDP in cadaveric lumbar discs [29]. In addition, its clinical effectiveness for patients with radiculopathy has been demonstrated in a randomized clinical trial [37]. According to practicing clinicians (personal communications) MCD procedure is commonly used to treat neck pain patients with radiating symptoms to the arms where discs in the lower cervical spine (C4-C5, C5-C6, C6-C7, and C7-T1) are involved. 

 This study was designed to measure IDP changes during a manual cervical distraction procedure in the lower cervical discs (C4-C5, C5-C6, C6-C7, and C7-T1). MCD is commonly performed in a prone position and is different from other traction procedures used in various studies [38–46]. In this study, longitudinal traction along the length of the spine with contacts at C5 and C6 was performed with the cadaver in the prone position. This position allowed contacting the posterior arch of the specified cervical vertebra (C5 or C6). This is substantially different than the standard supine, upright seated, or standing forms of spine traction that apply forces to the vertebral column with no localizing contact. 

Although the studied procedures may all be applied to patients in the clinical setting, most patients receive one, but not all, of these procedures. The majority of discogenic pain patients receive neutral traction or fixed flexion and traction. Few DCs use combined moving flexion and MCD. Patient tolerance guides selection of the specific traction procedure. Part of the study was to determine if there was additional or varied physiological benefit (drop in intradiscal pressure) when performing the traction alone or traction with fixed flexion or combining the flexion and traction simultaneously. Recovery time of two minutes was allowed between the different traction conditions. Previous biomechanical studies have used recovery times ranging from 15 seconds to 4 minutes [47–52]. To minimize testing time and tissue degradation we chose a recovery time of two minutes.

 IDP decreases were observed at all levels for DC 1 under neutral traction conditions. IDP increased at C7-T1 level for some of the DCs when the contact was at C6 level. During generalized traction, IDP at C7-T1 increased for most of the DCs. In general, DCs applied higher forces when contacting at C5 compared to C6 and higher forces during moving flexion compared to neutral and fixed flexion tractions (Table 3). DC4 applied the maximum traction force, while DC3 applied the least traction force. DC3 was the clinician in an academic research setting. All four DCs applied posterior-to-anterior (PA) force along with traction. The level of PA forces was higher for DC4, followed by DC1, and then DC2. DC3 had the smallest forces among all the DCs. All the DCs applied higher PA force when contact was at C6. Contact at C6 was more difficult due to the anatomical region, which may explain this finding. 

Traction forces used by the four DCs in our study (Table 3) were in the range reported by several other investigators using home traction application in clinical studies. Raney et al. [53] used traction forces of 23.2lbs (103 N), Young et al. [46] used 35lbs (156 N), Fater and Kernozek [38] used 13.6 Kg (133 N), Tsai et al. [54] used 10%–30% of body weight (47 N–141 N), and Forbush et al. [45] used traction forces of 9–13 kg (88 N–128 N). Young et al. used 5lbs (22 N) as a sham traction force for their control group [46].

All DCs had high intraclinician reliability on changes in IDP for the three sets of the procedure at all levels (Table 4). This suggests that MCD can be delivered consistently by practicing DCs as well as academic/research clinicians. The traction forces for two of the four DCs had high reliability, while the other two had moderate reliability. This suggests that some DCs may need training to deliver traction forces more consistently. We did not perform interclinician reliability because of the small sample for three of the DCs.

The hand contact position and force for each of the clinicians were likely different and may have influenced the lordosis of the cervical spine which, in turn, may have contributed to variations in the tractions forces. This could be one reason why the intraclass coefficients are smaller for two of the four DCs. The PA forces for these two DCs were higher. The decrease in intradiscal pressure can be induced by not only the applied traction forces but also the tensile forces in the intervertebral disc produced by increased lordosis. Thus, while the introduction of a lordosis may have decreased the reliability of the traction force delivered by a DC, the additive effect on the intradiscal pressure due to traction and lordosis may have improved the reliability of the intradiscal pressure change. 

Wu et al. [33] reported IDP changes during simulated spinal manipulations on 7 cadaveric cervical spine specimens [33]. They tested the cadavers in an upright position using an MTS machine with compressive load (100 N), traction load (200 N), flexion, and extension (10 deg., 20 deg.). During traction phase they reported mean pressure decreases of 75 KPa at C3-C4, 84 KPa at C4-C5, and 70 KPa at C5-C6. Li et al. [32] reported significant decreases in IDP during simulated traction loads of 150–200 N on a cervical spine in an upright (vertical) position. They also observed increases in IDP during rotation and concluded that traction followed by rotation is a safer manipulation. 

Our studies are based on prone traction as applied during clinical practice whereas the studies by Yi-Kai et al. and Wu et al. were based on vertical position simulated using a material testing system. The IDP decreases at C4-C5 and C5-C6 under manual cervical distraction in our study were comparable to the reported values by Wu et al. during the traction phase. The mean IDP decreases reported by Li et al. [32] at 200 N were much higher than observed in our study and those of the study by Wu et al. [33]. It is important to note that we did not measure pressure changes at C3-C4 and Yi-Kai et al. and Wu et al. did not report pressure changes at C6-C7 and C7-T1. In addition, the forces the forces used by our clinicians were smaller than the simulated forces used by Wu et al. [33] and Li et al. [32]. Traction forces of 200 N are much higher than the forces commonly used in clinical studies as well as our study.

Li et al. [32] and Wu et al. [33] obtained cadaveric specimens from the Chinese population while our specimens were drawn from the US population. In addition, Li et al. [32] specimens were male, 23–34 years old, and Wu et al. [33] specimens were male and female, 28–39 years of age. Cadaveric specimens in our study were male and female, 28-54 years of age. This could contribute to some differences in the observations. This could contribute to some of the differences in the results of our study compared to Li et al. [32] and Wu et al. [33]. 

Disc degeneration has an influence on changes in IDP. In our study, only two discs were found to have greater than Grade I degeneration. Hence we did not account for disc degeneration as a factor in our observations. 

### 4.1. Limitations

Unembalmed cadavers were used in this study and it is appreciated that active musculature during in vivo situations could alter the changes in the IDP. It is a standard practice in numerous biomechanical studies published in the literature to use human cadaveric spine specimens to assess the mechanical response of intervertebral discs in order to understand how the human spine may respond to physiologic loads (forces and moments) experienced during activities of daily living. Three of the four DCs in our study are in clinical practice and use this technique on a day-to-day basis, but they were not given instructions regarding control of maximum force application. Intervertebral discs were graded based on a single-lateral static view of video fluoroscopic images, which is not optimal. For future studies we will consider magnetic resonance images of the spine to grade the discs. We do not know of any better technology that can be used at this stage.

## 5. Conclusions

In this cadaveric study we observed decreases in IDP in the lower cervical spine during a chiropractic MCD procedure in prone position. Based on the maximum number of specimens DC1 has done, moving flexion and traction seem to reduce more IDP, followed by neutral traction, fixed flexion and tractions, and generalized traction. Although the doctors of chiropractic in this study demonstrated good intraclinician reliability, the magnitude of traction forces varied. Larger powered studies should be undertaken to determine if these decreases in IDP are significant depending on the doctor, contact location, and the different traction procedures. Also, the clinical significance of these differences is unknown.

## Figures and Tables

**Table 1:** Specimen demographics.

Spec.	Gender	Age	COD	Height (cm)	Weight (kg)	BMI
1	F	28	Glioblastoma	170	96.6	33.4
2	F	52	COPD	155	67.1	28.0
3	F	50	Multiple organ failure	160	86.2	33.7
4	M	43	Liver disease	180	108.9	33.5
5	M	43	Primary lateral sclerosis	185	72.6	21.1
6	F	52	Cardiac arrest	168	68.0	24.2
7	M	46	Primary myelofibrosis	155	72.6	30.2
8	F	34	Uterine cervix cancer	175	60.8	19.8
9	M	54	Mouth cancer	185	95.3	27.7

	Mean	**44.7**		**170.5**	**80.9**	**28.0**
	SD	8.8		12.1	16.4	5.3

**Table 2:** Summary of pressure decreases (kPa) for all DCs.

Contact location	DC1, *N* = 7		DC2, *N* = 4		DC3, *N* = 2		DC4, *N* = 3	
	Mean	SD	Mean	SD	Mean	SD	Mean	SD
Neutral traction								
C5								
C4-C5	**76.8**	67.6	**118.6**	138.3	**64.8**	—	**52.0**	70.6
C5-C6	**75.3**	52.2	**53.0**	44.6	**50.5**	—	**100.1**	31.4
C6-C7	**67.7**	47.6	**47.2**	23.1	**27.9**	—	**83.2**	5.1
C7-T1	**39.1**	47.8	**0.1**	66.9	**16.5**	—	**62.7**	2.7
C6								
C4-C5	**70.5**	68.7	**118.5**	103.5	**74.9**	32.3	**104.0**	90.8
C5-C6	**63.4**	51.4	**44.9**	25.1	**19.2**	27.1	**81.6**	79.1
C6-C7	**63.7**	38.9	**47.2**	49.9	**30.7**	17.0	**93.7**	4.6
C7-T1	**11.2**	64.5	−**17.5**	78.9	−**30.2**	72.1	−**0.9**	95.7

Fixed flexion and traction								
C5								
C4-C5	**46.5**	52.4	**91.6**	131.3	**13.3**	—	**4.7**	9.5
C5-C6	**41.9**	33.3	**31.9**	48.6	**27.3**	—	**48.7**	44.5
C6-C7	**54.9**	34.8	**35.7**	36.6	**8.9**	—	**51.2**	61.0
C7-T1	**10.5**	53.4	**14.6**	42.7	**10.7**	—	**69.2**	47.4
C6								
C4-C5	**54.8**	65.8	**97.0**	102.5	**28.7**	12.1	**2.3**	65.1
C5-C6	**48.7**	38.9	**20.0**	23.1	**10.2**	14.5	**47.6**	41.5
C6-C7	**59.8**	34.9	**12.8**	76.6	**23.3**	19.1	**42.0**	23.7
C7-T1	−**7.0**	69.0	−**31.8**	63.8	−**23.4**	56.2	−**22.5**	51.6

Moving flexion and traction								
C5								
C4-C5	**82.8**	56.1	**112.5**	150.2	**92.8**	—	**36.2**	60.8
C5-C6	**68.1**	65.1	**41.4**	46.8	**75.2**	—	**91.2**	21.5
C6-C7	**84.9**	64.5	**38.9**	38.1	**37.3**	—	**79.9**	25.8
C7-T1	**41.5**	55.3	**20.4**	55.9	**35.7**	—	**57.6**	27.6
C6								
C4-C5	**86.2**	49.3	**168.7**	211.1	**114.7**	47.4	**118.7**	116.9
C5-C6	**65.8**	55.5	**55.1**	48.7	**31.8**	45.0	**87.0**	82.8
C6-C7	**94.3**	64.0	**33.0**	16.1	**64.0**	56.7	**70.4**	53.0
C7-T1	**26.4**	52.2	−**58.1**	70.9	−**21.5**	62.7	−**19.2**	63.2

Generalized traction								
C4-C5	**43.0**	72.5	**118.6**	143.9	**94.9**	1.9	**51.0**	55.4
C5-C6	**58.6**	67.9	**85.7**	106.3	**48.6**	68.8	**98.4**	88.6
C6-C7	**53.1**	33.7	**60.5**	59.0	**30.9**	16.1	**146.1**	30.8
C7-T1	−**29.4**	80.9	−**15.3**	68.0	−**16.6**	13.9	—	—

Generalized traction fixed flexion								
C4-C5	**66.0**	83.3	**51.7**	77.0	**34.2**	31.4	**23.0**	46.3
C5-C6	**38.6**	59.8	**45.1**	70.1	**15.2**	21.7	**50.6**	49.3
C6-C7	**73.6**	82.5	**67.1**	68.9	**69.8**	81.1	**103.2**	60.9
C7-T1	−**23.7**	50.6	**0.2**	68.0	−**8.1**	6.3	**11.9**	46.1

Note that, for DC3, one of the specimens he tested did not have C5 as a contact location; therefore, no SD could be calculated, negative numbers represent increases in IDP.

**Table 3:** Summary of forces (*N*) for all DCs.

Contact location	DC1, *N* = 8		DC2, *N* = 4		DC3, *N* = 3		DC4, *N* = 4	
	Mean	SD	Mean	SD	Mean	SD	Mean	SD
Neutral traction								
C5								
Traction force	**88.6**	9.3	**73.3**	14.2	**56.2**	19.9	**101.8**	8.2
Lateral force	−**11.3**	4.5	−**5.3**	1.3	−**0.5**	1.5	−**12.9**	4.6
PA force	**30.2**	9.2	**11.8**	3.6	**20.2**	14.5	**39.4**	3.1
C6								
Traction force	**72.6**	17.0	**78.0**	14.7	**52.1**	29.9	**94.6**	23.3
Lateral force	−**9.4**	2.1	−**3.3**	4.3	−**4.1**	2.2	−**8.9**	13.1
PA force	**34.0**	10.2	**16.7**	8.0	**24.6**	14.4	**56.4**	9.2

Fixed flexion and traction								
C5								
Traction force	**87.4**	12.6	**83.5**	21.1	**56.7**	35.1	**109.6**	19.1
Lateral force	−**12.4**	4.6	−**4.3**	6.5	−**4.6**	2.6	−**17.8**	7.5
PA force	**42.1**	9.8	**15.1**	3.9	**30.3**	22.2	**60.4**	9.4
C6								
Traction force	**72.5**	16.6	**77.2**	21.1	**54.2**	27.3	**99.1**	22.9
Lateral force	−**9.6**	3.4	−**3.6**	4.5	−**3.8**	2.8	−**14.3**	8.1
PA force	**45.9**	8.6	**27.5**	9.8	**28.1**	14.4	**61.6**	5.1

Moving flexion and traction								
C5								
Traction force	**101.7**	16.1	**127.6**	38.3	**84.8**	38.7	**107.1**	9.0
Lateral force	−**9.6**	3.5	−**11.8**	5.6	−**2.2**	2.2	−**13.9**	7.5
PA force	**53.1**	16.0	**34.6**	9.2	**21.2**	12.9	**63.6**	9.9
C6								
Traction force	**90.2**	24.8	**119.2**	37.3	**79.9**	37.4	**91.4**	12.4
Lateral force	−**3.5**	9.7	−**10.4**	12.2	−**2.8**	4.8	−**8.4**	7.9
PA force	**56.1**	12.1	**55.0**	18.5	**62.4**	45.5	**82.6**	10.2

**Table 4:** Intraclass correlation coefficients assessing IDP changes and traction force intraclinician reliability.

	C4-C5 pressure	C5-C6 pressure	C6-C7 pressure	C7-T1 pressure	Traction force
DC1	0.88	0.89	0.97	0.90	0.59
DC2	0.93	0.86	0.83	0.95	0.93
DC3	0.96	0.96	0.99	0.95	0.93
DC4	0.85	0.84	0.83	0.64	0.52
